# Isolated symmetrical bilateral basal ganglia T2 hyperintensity in carbon monoxide poisoning

**DOI:** 10.4103/0972-2327.44563

**Published:** 2008

**Authors:** S. Subhaschandra, W. Jatishwor, Th. Suraj

**Affiliations:** Department of Radiodiagnosis, Regional Institute of Medical Sciences, Imphal, Manipur, India; 1 Department of Neurology, Regional Institute of Medical Sciences, Imphal, Manipur, India

**Keywords:** Basal ganglia, carbon monoxide, T2- hyperintensity

## Abstract

Carbon monoxide poisoning is not uncommon during the winter months. To make a diagnosis, strong clinical suspicion and acumen, and history of the exposure are necessary. Many a time, the presenting complaints may fail to help reach a diagnosis, in the absence of history. Imaging plays a role in the diagnosis of brain injury with the characteristic features, which are correlated with the clinical profile. Isolated bilateral basal ganglia injury revealing T2 hyperintensity in MRI may be observed in acute carbon monoxide poisoning.

## Introduction

Carbon monoxide is produced by incomplete combustion of carbon based fuels and household fires. Carbon monoxide poisoning usually occurs during the winter months. Hypoxia, cellular asphyxia, ischemia and reperfusion injury are the pathological changes in carbon monoxide poisoning. Carbo monoxide combines reversibly with hemoglobin to produce COHb diplacing oxygen and reducing blood oxygen content. Carbon monoxide has an affinity of 200-300 times that of oxygen.[1]

There are variable presentations of carbon monoxide poisoning, ranging from asymptomatic or slight headache, dizziness, nausea, syncope, visual disturbance, confusion, seizure and coma. Carbon monoxide poisoning may even cause cardiopulmonary failure and death.[1] Recurrence of neurological and psychiatric symptoms preceded by a lucid interval of two to three weeks after the recovery of the acute stage may be observed and is known as delayed encephalopathy of carbon monoxide poisoning.[2] History of exposure to carbon monoxide, clinical suspicion and acumen are important for making the diagnosis. The level of blood COHb may be low or undetectable because of the time gap between the exposure and the presentation. Computerized tomography (CT) and/or magnetic resonance imaging (MRI) of the brain will indicate the characteristic and extent of the injury occurred in the brain, involving basal ganglia, substantia nigra, cerebral cortex, and the hippocampus. The extent of the lesion may be correlated with clinical severity.[3] In the absence of reliable history of exposure, the differential diagnosis includes viral illness, food poisoning, depression, TIA, CAD, and functional illnesses.[1]

We present a case with typical history and clinical presentation of carbon monoxide poisoning with the MRI revealing peculiar symmetrical isolated basal ganglia (globus pallidus) T2 hyper intensity. No other focal lesion or altered signal was seen in the other parts of the brain. The substantia nigra was not involved.

## Case Report

**Figure 1:**
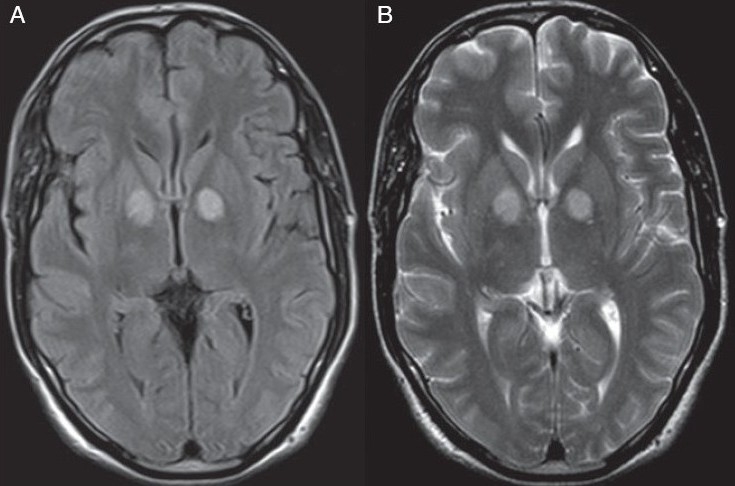
(A) FLAIR axial image showing bilateral symmetrical basal ganglia lesions (B) T2 weighted axial MR image showing bilateral symmetrical basal ganglia lesions

A 55-year-old male patient was found unconscious in a closed room, with marked hypotension (BP 80/40 mm Hg) and central cyanosis, early in the morning, in the month of February 2007. He was admitted with the clinical impression of CAD/stroke. On further enquiry, it was revealed that he slept in the room with burning charcoal heater. So the probability of carbon monoxide poisoning was considered. Blood gas analysis revealed elevated pCO2. Other bochemical tests like kidney function test(urea,creatinine,sodium, potassium) and liver function test were within normal limits. He was managed conservatively and he regained consciousness the next day, though he was delirious. Non contrast CT scan was done the next day, which was reported normal. He was sent for MRI examination on the fourth day, which revealed bilateral symmetrical T2 hyperintense signal involving the globus pallidus [Figures 1A, 1B]. It was iso-intense in T1 weighted images. No other focal signal change was seen. The patient was fully recovered and discharged on the 15^th^ day. There was no history or clinical features of delayed encephalopathy when the patient came for follow up after one month. No follow up/check MRI was performed.

## Discussion

In carbon monoxide poisoning, the gas crosses the alveolar capillary membrane and binds strongly with heme containing compounds. The toxicity is due to hypoxia and carbon monoxide mediated damage at the cellular level.[4] Globus pallidus and pars reticulata of substantia nigra are the regions of the highest iron content in the brain. Carbon monoxide directly binds to heme iron in these two regions. This direct histotoxicity explains selective vulnerability of the globus pallidus and substantia nigra to carbon monoxide poisoning.[8] Periventricular white matter, subcortical white matter, temporal lobe including the hippocampus, thalamus, and cerebellum are the other areas where there is usually brain injury, in case of carbon monoxide poisoning.[5,6,7]

Clinical features of acute carbon monoxide poisoning are variable, ranging from asymptomatic or mild headache to coma and seizure or cardiopulmonary dysfunction and death.[1] Delayed encephalopathy of carbon monoxide poisoning develops after a lucid interval of two to three weeks.[2] The symptoms consist of a triad of mental deterioration, urinary incontinence, and gait disturbance, as well as other neuropsychiatric manifestations.[2,3]

Magnetic resonance imaging is the modality of choice for imaging and assessing the cerebral lesion. Imaging may be normal in early acute carbon monoxide poisoning.[6] In acute carbon monoxide poisoning, the abnormalities are detected as T2 hyperintensities. Lesions are in the basal ganglia involving the globus pallidus, putaman, caudate nucleus with other areas such as periventricular white matter, subcortical white matter, cerebral cortex, medial temporal lobe (hippocampus) and cerebellum.[6] The most common finding in the study of Durak *et al*., was bilateral symmetric hyperintensity of the white matter, which was more significant in the centrum semiovale, with relative sparing of the temporal lobes and anterior parts of the frontal lobes on T2-weighted and FLAIR images.[3] The extent of the lesion as seen MRI is correlated with the clinical picture and severity as patients with severe carbon monoxide poisoning may develop persistent MRI changes. An MRI is also useful for follow up cases for residual lesions in chronic carbon monoxide poisoning.[3,8] Cerebral atrophy, atrophy of the cerebellum vermis and corpus callosum, and T2 hyperintensity of the centrum semiovale are seen in cases of chronic carbon monoxide poisoning, with neuro-psychiatric sequelae.[3] Other sequences of Magnetic Resonance Imaging like Diffusion Weighted Imaging(DWI) and MR Sprectroscopy are also useful. Diffusion weighted imaging shows diffuse hyperintensity in periventricular white matter and semiovale, splenium of corpus callosum, internal capsules, and brainstem, with moderately decreased apparent duffusion co-efficient (ADC) values. In the globus pallidus, the ADC values are rather increased, with low signal intensities on DWI.[2,4] The MRS demonstrates decreases n-acetyl aspartase in bilateral basal ganglia.[8]

Spin echo T1, Spin echo T2, and FLAIR sequences were performed in the case for the 55-year-old patient. Bilateral symmetrical T2 hyperintensity in the globus pallidus of the basal ganglia was observed. No other focal lesion or altered signal was seen in the cerebral cortex. The lesion was iso-intense in T1 weighted images. A peculiar feature in this index case was that there was no other focal lesion, except the bilateral symmetrical globus pallidus lesions. The substantia nigra was normal. Since serial or follow up MR scan was not performed, it may not be true to conclude that no other area of the brain was involved. However, it may be observed that only the globus pallidus of the basal ganglia may be affected symmetrically, in early cases of acute carbon monoxide poisoning.
